# A Mixture Model and a Hidden Markov Model to Simultaneously Detect Recombination Breakpoints and Reconstruct Phylogenies

**DOI:** 10.4137/ebo.s2242

**Published:** 2009-06-25

**Authors:** Bastien Boussau, Laurent Guéguen, Manolo Gouy

**Affiliations:** Université de Lyon; université Lyon 1; CNRS; UMR 5558, Laboratoire de Biométrie et Biologie Evolutive, 43 boulevard du 11 novembre 1918, Villeurbanne F-69622, France. Email:boussau@biomserv.univ-lyon1.fr

**Keywords:** molecular phylogeny, recombination, maximum likelihood, PhyML

## Abstract

Homologous recombination is a pervasive biological process that affects sequences in all living organisms and viruses. In the presence of recombination, the evolutionary history of an alignment of homologous sequences cannot be properly depicted by a single bifurcating tree: some sites have evolved along a specific phylogenetic tree, others have followed another path. Methods available to analyse recombination in sequences usually involve an analysis of the alignment through sliding-windows, or are particularly demanding in computational resources, and are often limited to nucleotide sequences. In this article, we propose and implement a Mixture Model on trees and a phylogenetic Hidden Markov Model to reveal recombination breakpoints while searching for the various evolutionary histories that are present in an alignment known to have undergone homologous recombination. These models are sufficiently efficient to be applied to dozens of sequences on a single desktop computer, and can handle equivalently nucleotide or protein sequences. We estimate their accuracy on simulated sequences and test them on real data.

## Introduction

Homologous recombination is a process through which genes descending from a common ancestor exchange parts of their sequence. Consequently, sequences having undergone recombination will display two different histories: one history for one part of their sequence, affected by the recombination event, and one history for the other part. If the recombining genes have been parts of different lineages long enough prior to this recombination event, the difference in the histories of the recombining and non-recombining parts of the gene may translate into topological incongruencies between their respective phylogenies.

If one applies classical phylogenetic methods to an alignment that has undergone recombination, only one tree will be recovered, with no guarantee that this tree corresponds to the part of the sequence whose history has been affected by the recombination event, the other part, or any of these two. Several methods have been developed to try and detect recombination in alignments;^1^,^2^ such methods can therefore be used prior to phylogenetic analysis to see whether it is meaningful to describe the history of an alignment by a single bifurcating tree. In cases where no recombination has been detected, the subsequent analysis is classical phylogenetics. In cases where recombination has been detected, there are few methods available that can analyse an alignment and precisely predict both the recombination breakpoints and the evolutionary histories found in the alignment.

If we put aside methods based on sliding windows, that cannot precisely pinpoint the recombination breakpoints, a few groups have proposed methods to unveil both the recombination positions and the phylogenetic trees. In 2000, Mcguire et al^3^ inspired by the work of Felsenstein and Churchill,^4^ proposed a method based on a hidden Markov model (HMM) in which the hidden states were the phylogenetic trees themselves. Therefore, a transition between the states ought to be a recombination breakpoint. However, this first attempt was prone to misinterpreting rate heterogeneity as recombination events. Husmeier subsequently built upon this model to deal with heterogeneities in site evolutionary rates^5^ by superimposing another HMM whose states correspond to evolutionary rates: therefore two kinds of transitions are allowed along the alignment, a transition between topologies, indicative of recombination, and a transition between rates. Unfortunately, all these methods are computationaly demanding, and can only be applied in cases where the space of tree topologies is very limited, as all topologies need to be given *a priori.* Lastly, Kedzierska and Husmeier^6^,^7^ proposed a hybrid approach in which a sliding window is first applied to the alignment to build phylogenetic tree distributions along the alignment. Then, a HMM is run on the alignment, with its hidden states being the tree distributions themselves. This approach allows to handle a larger number of sequences than the previous ones, but is also probably less accurate in breakpoint detection, because the topology distributions are built from small arbitrary windows, which may not correspond to the true recombination structure of the alignment.

In 2002, Suchard and co-workers^8^ proposed a Bayesian multiple-changepoint model to detect recombination, and further improved it by adding a second changepoint process to account for changes in the substitutional process.^9^,^10^ This sophisticated method however also suffers from its computational requirements. In fact, both this method and those of Husmeier, Wright and co-workers have been implemented to only deal with DNA sequences, and cannot be used with large numbers of sequences.

However, the detection of recombination should not be limited to recently diverged sequences. When protein-coding sequences have diverged a long time ago, synonymous sites of the nucleotide sequence may be saturated, so that it becomes advisable to resort to amino-acid sequences. In such conditions, none of the previously described methods can be used.

Most recently, Pond and co-workers developped GARD,^11^,^12^ a software able to detect recombination with any type of alphabet. This program estimates the phylogenetic trees, the number of recombination breakpoints and their positions in a maximum likelihood framework. To do so, it tries different numbers of breakpoints, and for each number, uses a genetic algorithm to estimate the best breakpoint positions. During this procedure, phylogenetic trees are estimated with the Neighbor-joining algorithm,^13^ and the best number and positions of breakpoints are chosen according to the Akaike criterion. This considerable task can be achieved efficiently through a parallelised architecture, which can be run on a cluster of computers.

In this article, we present two new methods to uncover the recombination structure of a protein or nucleotidic alignment, that can be easily and efficiently run on a desktop computer. The first method is based on a Mixture Model (MM), and the second is based on a phylogenetic Hidden Markov Model (Phylo-HMM). We begin by introducing the mathematics behind these models, shortly explain how these were implemented, and finally proceed to test them on both simulated and real alignments. We discuss the merits and limits of our methods and propose a few refinements.

## Computing the Likelihood of a single Tree

**Figure 1:**
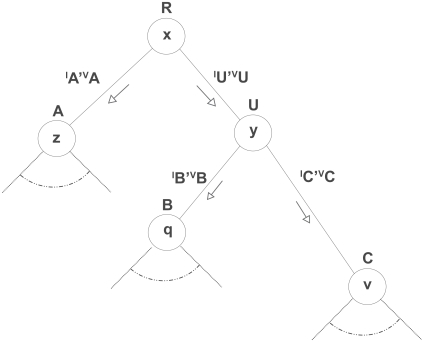
Example rooted tree for likelihood computation.

We first explain how one computes the likelihood of a phylogenetic tree^14^ with nucleotide or protein sequences using the following example (Fig. 1).

Most commonly, sites are supposed to evolve independently of each other: a site does not depend on its neighbors’ states but only on its past state. As a consequence, the likelihood of a tree for a whole sequence is the product of all the likelihoods obtained from single sites.

The likelihood *L_s_*_,_*_τ_* of the tree *T* given in Figure 1 for a single site *s* is computed as follows:

(1) $$\begin{array}{l} L_{s,T}=\frac{1}{\left|\Gamma \right|}\sum_{g\in \Gamma }[\sum_{x\in \Omega }[P(R=x)\\ \times \sum_{z\in \Omega }\left[P_{xz}(l_{A},g,\upsilon_{A})L_{s,low(RA)}(A=z)\right]\\ \times \sum_{y\in \Omega }(P_{xy}(l_{U},g,\upsilon_{U})\\ \times \sum_{q\in \Omega }\left[P_{yq}(l_{B},g,\upsilon_{B})L_{s,low(UB)}(B=q)\right]\\ +\sum_{\upsilon \in \Omega }\left[P_{y\upsilon }(l_{C},g,\upsilon_{C})L_{s,low(UC)}(C=\upsilon )\right])]] \end{array}$$

where *P_xy_*(*l_A_*, *υ_A_*) is the probability for base *x* to change into base *y* along a branch of length *l_A_*, with rate category *g* from the Γ distribution and other evolutionary parameters *υ_A_*, *P*(*R* = *x*) is the probability to have base *x* at the root *R*, and Ω is the set of possible states (for instance, Ω = {*A*, *T*, *C*, *G*} in case of a DNA alignment); *L_s_*_,_*_low_* _(_*_RA_*_)_(*A* = *z*) is the lower conditional likelihood of observing the data downstream from branch *R* → *A* conditionally on the underlying subtree and on having base *z* at node *A.* Note that computing the likelihood of a site when using a distribution over the evolutionary rates amounts to averaging the likelihoods of the site obtained when using each evolutionary rate in turn.

## Computing the Likelihood with a Mixture Model on Trees

As for the likelihood of a model where different rates are allowed, one can compute the likelihood of a model where one allows different trees. Consequently, to get the likelihood of a model whose parameters of interest are the trees that best describe the alignment, one can take at each site the average over the likelihoods obtained with each one of the trees that are considered.

This is summed-up in the following formula for the likelihood of a single site, where *T* represents the set of trees *τ* currently in use, and |*T*| the number of trees in *T*:

(2) $$L_{s,T}=\frac{1}{\left|T\right|}\sum_{\tau \in T}L_{s,\tau }$$

with such a formula, both across-site rate heterogeneity and topology heterogeneity are taken into account, respectively by the gamma distribution and the Mixture Model on topologies. Once the likelihood of a Mixture Model over trees has been computed and maximized, it is possible to predict *a posteriori* the most likely tree for a given site (see below). This possibility can be used to uncover the recombination structure in an alignment.

### Toy example: it is possible to optimize the topologies with a Mixture Model on trees

In a setting where we search for |*T*| trees *τ* that describe an alignment, we try to find the set of |*T*| trees whose likelihood as computed above in Eq. 2 is maximal. The object that is looked for is the set *T* itself. In principle, a set of |*T*| distinct trees can have a higher likelihood than that of any single tree, as can be seen in this toy example, where |*T*| = 4, with 4 sites:

**Table:** 

Topologies	Site 1 likelihood	Site 2 likelihood	Site 3 likelihood	Site 4 likelihood
Topology 1	10^−2^	10^−4^	10^−4^	10^−4^
Topology 2	10^−4^	10^−2^	10^−3^	10^−4^
Topology 3	10^−4^	10^−4^	10^−2^	10^−4^
Topology 4	10^−4^	10^−4^	10^−4^	10^−2^

In this example, the most likely topology is *Topology* 2, with a log-likelihood of *log*(10^−4^ × 10^−2^ × 10^−3^ × 10^−4^) = −13. However, if one uses a Mixture Model on tree sets containing 4 trees, this does not simply result in the same *Topology* 2 topology being found in the 4 trees. Indeed, as the average over the likelihoods of each topology is computed for each site, one obtains the following log-likelihood:

$$\begin{array}{l} log(L_{T})=log(\frac{10^{-2}+3\times 10^{-4}}{4}\times \frac{10^{-2}+3\times 10^{-4}}{4}\\ \times \frac{10^{-2}+2\times 10^{-4}+10^{-3}}{4}\times \frac{10^{-2}+3\times 10^{-4}}{4})\\ \approx -10.3 \end{array}$$

It is thus more likely on this example to use 4 different trees rather than a single tree. However, had the alignment been homogeneous, this model could have resulted in the same tree repeated 4 times, possibly with branch lengths differing between trees.

This example shows that in case of an alignment altered by a recombination event, a set of |*T*| trees can be optimized to best account for the sequence evolution with a Mixture Model: it is not necessary that the tree topologies are specified before the search for the recombination breakpoint is undertaken.

## A Phylogenetic Hidden Markov Model to Detect Recombination

The Mixture Model described above fails to account for an important property of the alignment: it is expected that the topology that best describes a given site has a high probability of properly describing the neighboring sites. Thus there is a dependency between sites, that can be modelled through the use of a Hidden Markov Model, whose hidden states are the topologies themselves. This model therefore belongs to the family of Phylo-HMMs. The rate heterogeneity is taken into account through a mixture model on rates, through the commonly used gamma distribution.

### Computing the likelihood with the Phylo-HMM

The likelihood of the Phylo-HMM can be computed with the forward algorithm, as already explained in the phylogenetics framework by Felsenstein and Churchill.^4^ We rapidly go through this algorithm here.

The algorithm starts from one end of the alignment and finishes at the other end; arbitrarily, we will start by the beginning of the alignment, at site 1, and end at site *n.* We suppose that individual site likelihoods have been already computed for all the trees. We note as *L*_1,_*_τ_* the likelihood obtained with Felsenstein’s pruning algorithm at site 1 for the tree *τ*. The likelihood of the alignment up to site *k* with tree *τ* associated to site *k* is denoted *L_τ_*^(^*^k^*^)^. The transition probability of going from tree *τ* at site *k* to tree *τ*′ at site *k* + 1 is written *P_τ_*_,_*_τ_*_′_. We define as |*T*| the total number of trees in the set *T*.

At the first site, the likelihood of the alignment up to site 1, given that site 1 has tree *τ* is simply the likelihood of tree *τ* for the site 1:

$$L_{\tau }^{\left(1\right)}=L_{1,\tau }$$

At the second site, the likelihood of the alignment up to site 2, given that site 2 has tree *τ*′

$$L_{\tau^{\prime }}^{\left(2\right)}=L_{2,\tau^{\prime }}\times \sum_{\tau \in T}P_{\tau ,\tau^{\prime }}L_{\tau }^{\left(1\right)}$$

This formula suggests a recursive scheme:

$$L_{\tau^{\prime }}^{\left(k+1\right)}=L_{k+1,\tau^{\prime }}\times \sum_{\tau \in T}P_{\tau ,\tau^{\prime }}L_{\tau }^{\left(k\right)}$$

The first part of the formula before the multiplication symbol is the classical likelihood of a tree for site *k*, which can be obtained through Felsenstein’s pruning algorithm^14^ as in Equation 1. The dependency between sites is introduced through the second part of the formula. At the end of the alignment, at site *n*, the total likelihood of the alignment given the set of trees *T* is computed as follows:

(3) $$L_{Phylo-HMM}=\sum_{\tau \in T}\frac{1}{\left|T\right|}\times L_{\tau }^{\left(n\right)}$$

In our model, the transition probability of going from tree *τ* at site *k* to tree *τ*′ at site *k* + 1, *P_τ_*_,_*_τ_*_′_ is defined as follows, with the help of the autocorrelation parameter *λ*:

$$P_{\tau ,\tau^{\prime }}=\lambda \delta_{\tau ,\tau^{\prime }}+\frac{1-\lambda }{\left|T\right|}$$

Here, δ*_τ_*_,_*_τ_*_′_ is the Kronecker delta function, which is 1 when (*τ* = *τ*′) and 0 otherwise. This means that at any site, there is a probability 1−*λ* that another tree is drawn for the next site, with the possibility that the same tree is drawn again.

Since one can compute the likelihood of the alignment with the Phylo-HMM, all parameters can be estimated in the maximum likelihood framework (or in a Bayesian framework). Therefore in our program, both the trees (topologies, branch lengths, parameters of the models) and the parameter *λ* are estimated by optimizing the likelihood as computed in equation 3, through the same algorithm as PhyML^15^ for common parameters, and through Brent’s numerical optimization algorithm^15^ for the autocorrelation parameter *λ*.

### Exploring the space of tree topologies with a mixture model on trees or with a phylogenetic hidden Markov model

The problem of optimizing |*T*| trees simultaneously is different from the problem of optimizing a single topology |*T*| times. At any given time, a topology is to be optimized *taking into account the other topologies.* Indeed, if each topology were optimized independently of the other topologies, the result would be |*T*| identical trees: this would have been equivalent to solving the single tree optimization problem |*T*| times, in parallel.

**Figure 2:**
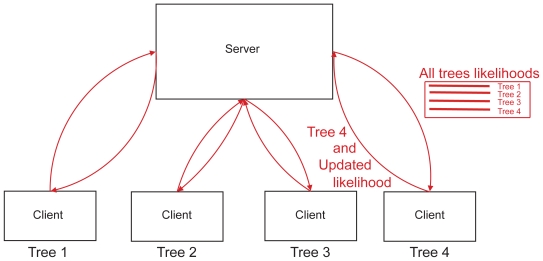
Client-Server architecture to efficiently find a set of topologies that best describe the alignment.

A parallel algorithm based on a client-server architecture, as described in Figure 2, allows to acknowledge the dependencies between topologies.

The server exchanges data with clients. For each set of communications between the server and a client, one red arrow corresponds to the sending by the server to the client of a matrix containing all the site likelihoods for all the topologies, and the other one corresponds to the sending by the client to the server of an optimized likelihood vector.

In this algorithm, each client is given a topology, which it tries to refine through commonly used tree search algorithms. However, while in common algorithms such as PhyML the client would simply try to maximize the likelihood of the topology, here it needs to maximize the likelihood of the MM or of the phylo- HMM as a whole, by only modifying the topology it has been given, while taking into account the other topologies. For instance, in the Mixture Model, the likelihood function each client tries to maximize thus is $L_{Tree\;mixture}=\Pi_{s}\frac{1}{\left|T\right|}\Sigma_{\tau \in T}L_{s,\tau }$, which implies that each client needs vectors of site likelihoods obtained from the other clients. The dependency between topologies is only taken into account through a shared matrix of likelihood vectors.

The algorithm has been summed up in the pseudo-code below.

**Algorithm 1** Searching for the most likely set of trees *T*.

likelihood_threshold= 1e-6

MAXIMUM= 1e6

|*T*| = 2

if (server) {

 get alignment aln

 set_of_trees T = Generate(|T|,aln)

 Create |*T*| clients

 send_all alignment

 send trees T

 likelihood_matrix = receive_all_likelihood_vectors()

 oldlk= compute_likelihood(likelihood_matrix)

 send_all(likelihood_matrix)

 diff= MAXIMUM

 while (diff>likelihood_threshold) {

  receive(likelihood_vector)

  update(likelihood_matrix)

  newlk= compute_likelihood(likelihood_matrix)

  diff= newlk – oldlk

  oldlk= newlk

  send_all(likelihood_matrix)

 }

 send_all(stop_signal)

 output_server_results

}

else if client {

  receive alignment

  receive tree

  compute_likelihood

  send(likelihood_ vector)

  receive(likelihood_matrix)

  while (not stop_signal)

  {

   optimize(tree, likelihood_matrix)

   send(likelihood_ vector)

 }

 output _client_results

 }

At the beginning of the program, the number of topologies to consider needs to be set, as this algorithm is not able to estimate the appropriate number of trees |*T*| to consider to describe the history of an alignment; in the pseudo-code above, it has been set to 2. In practice, setting this parameter should hardly be a problem, as a gene sequence should not harbour more than two (detectable) different evolutionary histories; it is however possible to specify more than two topologies to be searched for in a single alignment. At the begining of the algorithm, the function “Generate” divides the alignment in |*T*| equal parts and builds a BIONJ^16^ tree for each part. This results in |*T*| trees used as starting topologies for the bulk of the algorithm (alternatively, the user can also provide |*T*| starting trees). Each client then receives the alignment and a tree it is in charge of, computes the likelihood of this topology, and returns a vector of site likelihoods to the server. The server assembles all vectors into a matrix, that is sent to all clients. Each client subsequently modifies the specific tree it is in charge of, in order to maximize, *L_Tree mixture_* or *L_Phyio-HMM_*. Periodically, it sends an updated vector of site likelihoods to the server, which updates the likelihood matrix containing all likelihood vectors. This updated matrix is subsequently sent to all clients, so that they continue optimizing their topologies acknowledging the most recent changes in other topologies. In practice, communications between the server and the client are asynchronous, so that slowly-computing clients do not slow down the other clients. For the Phylo-HMM, the auto-correlation parameter *λ* is also exchanged between the server and the clients, and optimized by the server every ten times it receives a likelihood vector from one of its clients.

This algorithm has been implemented to function with both the MM and with the Phylo-HMM (where the autocorrelation parameter *λ* is exchanged between the server and clients, and periodically optimized by the server) in the PhyML-Multi program, based on PhyML v.2.4.4 code.^15^ This program can take advantage of a multi-processor or multi-core machine, by dispatching clients in charge of trees to different processors. It has been compiled and tested on Linux machines and can be downloaded at: http://pbil.univ-lyon1.fr/software/phyml_multi/

As a result, each client outputs an optimized topology, and the server outputs the matrix containing site likelihoods computed with each topology. If there have been recombination events in the history of the alignment, there should be stretches of sites whose most likely topology is the same. Through segmenting the matrix of site likelihoods, one should be able to uncover these stretches of sites with a common history. The Phylo-HMM can directly output a most likely segmentation; on the other hand, the Mixture Model does not provide such a segmentation.

### Partitioning the matrix of site likelihoods output by the mixture model

#### Methods to partition an alignment

Common approaches to segmentation involve the use of sliding windows, Hidden Markov Models or of the Maximum Predictive Partitioning algorithm (MPP algorithm).^17^,^18^ We have chosen not to use sliding windows, as the fixed size of the sliding window does not allow to precisely pinpoint the recombination events. Both the MPP algorithm and the HMM approach rely on a statistical approach to segment a sequence: given a set of models, they infer the most likely partitioning of the sequence into these models. In our case, the models are the trees themselves, and the sequence is the alignment. For each model, the site likelihoods have been previously computed by the MM or the Phylo-HMM. The partitioning of the alignment therefore is done according to these site likelihoods.

The Phylo-HMM approach permits to directly estimate a partitioning, which depends upon the transition probabilities between models. These transition probabilities can be estimated with the Baum-Welch algorithm.

The MPP algorithm on the other hand does not require that transition probabilities are set, but simply uses the matrix of site likelihoods as input for partitioning. More precisely, the MPP algorithm computes successively the most-likely partitions in at most *k* segments of a sequence, for all *k* from 1 to a given *n*, given a set of Markovian models. It is a modification of the partitioning algorithm of Bellman,^19^ adapted to the computing of the likelihood of Markovian processes. Let *L_t_*(*i*) be the likelihood of tree *t* at site *i.* If *M_k_*_,_*_t_*(*i*) is the likelihood of the most-likely partition in *k* segments of the alignment up to site *i* given that site *i* has tree *t*, and *M_k_*(*i*) is the likelihood of the most-likely partition in *k* segments of the alignment up to site *i*:

$$\begin{array}{l} M_{1,t}(i)=L_{t}(1)\times \cdots \times L_{t}(i)\\ M_{k+1,t}(i)=L_{t}(i)\times \text{max}(M_{k}(i-1),M_{k+1,t}(i-1))\\ M_{k}(i)=\underset{t}{\text{max}}\;M_{k,t}(i) \end{array}$$

We can see that the most-likely partitioning of the alignment in *k* segments *M_k_*(*n*), can be computed from the likelihoods of the models at each site in a time proportional to the product of the number of trees and the number of sites.

The MPP algorithm thus provides most likely partitionings in *k* segments, *k* ∈ [l, *n*]. In the end the user is faced with a range of most likely partitionings, among which a choice is to be made according to some criterion.

#### Estimating the number of segments with the MPP algorithm

As the number of segments increases, the likelihood of the segmentation generally also increases, not necessarily because adding a segment reveals a significant property of the alignment, but also because adding a segment may permit it to better fit a non-significant heterogeneity in a particular part of the alignment. In other words, the improvement in likelihood observed when the number of segments increases is due to the fitting of the “noisy” part of the signal rather than the meaningful part.

Such non-significant gains in likelihoods can also be seen in alignments where sites have been randomly swapped, erasing the meaningful signal of the recombination structure, but where nonsignificant heterogeneities are expected to be found simply by chance. Therefore the comparison between the true alignment and randomized versions of the alignment permits to distinguish improvements in the likelihood of a partitioning due to the uncovering of a homogeneous segment coming from a past recombination event from “noise” improvements in the likelihood, due to the fitting of non-significant heterogeneities.

To get an estimate of the number of segments in an alignment, the following protocol is thus applied, for each number *i* of segments in [1; *n*], with *n* defined *a priori* by the user:

- the likelihood *L* of the most likely partitioning in *i* segments is computed using the MPP algorithm
- the matrix of site likelihoods is randomized 50 times by swapping columns of site likelihoods (which is equivalent to swapping sites in the alignment), and for each of these 50 replicates, the likelihood of the most likely partitioning is computed using the MPP algorithm; the average *l̄* of these 50 likelihood replicates is computed
- the value L* = *L*/*l* is computed and used as a normalized likelihood for the partition in *i* segments.

In the end, all normalized likelihoods can be compared; the partitioning with the highest normalized likelihood is considered as the most reasonnable partitioning.

## Tests of the Mixture Model and the phylo-HMM Model

In our Phylo-HMM, HMM segment lengths follow a geometric law of parameter (|T|−1)×(1−*λ*)/|T|. The autocorrelation parameter *λ* is thus in direct relation with segment length. In cases where the alignment has undergone recombination and the two parts of the alignment are of very different size, the fact that all states share the same value for the autocorrelation parameter may pose a problem, as the lengths of the segments are highly variable. The MPP approach does not need a parameter for determining segment length, and may therefore produce different results from the HMM segmentation. The Phylo-HMM approach and the MM + MPP approach may therefore complement each other, each having defaults that the other does not have. This suggests that both approaches should be used in parallel, and their results compared. In this purpose, we used simulations.

### Simulation procedure

The first 100 trees from the PhyML test set^15^ were selected. These trees contain 40 leaves, were designed to resemble real-life datasets and should therefore provide an appropriate test-set. An alignment affected by a recombination is an alignment in which one part is best described by a particular tree, and the rest by another tree. In the most difficult instances, the two trees corresponding to the two parts of the alignment differ by a single clade whose position is in one place in the first tree, and another place in the other tree. To obtain such pairs of trees, each of the 100 trees was subjected to a Subtree Prune and Regraft operation (SPR), in which a subtree is detached from the tree and attached in another position. This yielded pairs of trees separated by one recombination event, with Robinson and Foulds distances^20^ ranging from 2, when the SPR regrafted the pruned subtree very close to its original position, to 30, when the pruned subtree was regrafted far from its original position. Alignments harbouring a recombination event were simulated by evolving a portion of an alignment according to one of the 100 trees and the rest of the alignment according to the same tree modified by the SPR. For each alignment, there was only one breakpoint position. For each pair of trees, nine 1000-nucleotide alignments were simulated with *k* sites according to one tree and 1000—*k* sites according to the other tree, with *k* taking the values 100, 200, 300, 400, 500, 600, 700, 800, 900. Seq-Gen^21^ was used to simulate sequences, with the GTR model^22^ and a continuous gamma rates across site distribution with parameter alpha set to 0.8.

### Reconstruction of the recombination structure with the Mixture Model and the hidden Markov Model

Both the Mixture Model and the Hidden Markov Model were applied to the simulated datasets. The number of trees were set to two for both examples, as none of the programs is able to estimate the right number of trees to consider to faithfully describe an alignment. The evolutionary model used was HKY^23^ with a gamma distribution discretized in four classes to account for accross site rate variation. The reconstruction model therefore does not exactly correspond to the simulation model, as would be the case in a realistic setting where sequences have evolved according to an unknown and complex process.

### Ability to detect the right number of segments

**Figure 3:**
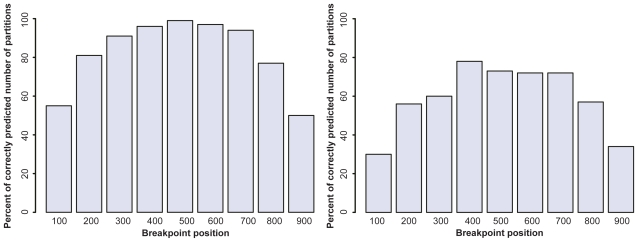
Ability of the Phylo-HMM (left) and the Mixture Model (right) and to detect the number of segments in simulated alignments.

The reconstruction models should detect two parts in the alignment, each part corresponding to different tree topologies. Although the algorithm was constrained to look for a set of 2 trees, it may find that the alignment is broken in more than 2 segments, with the first predicted to have evolved according to tree 1, the second segment according to tree 2, the third segment according to tree 1 again, etc … Figure 3 shows that both models have a recovery rate of the right number of segments that is dependent upon the position of the breakpoint. If the breakpoint is too close to the begining or the end of the alignment, the recovery rate is lower than if the breakpoint is more central. This is likely because lengths such as 100 or 200 nucleotide sites contain too little information to properly reconstruct a tree topology. Such values may therefore represent the statistical limit below which our models cannot detect recombination. The Phylo- HMM is more efficient than the MM in all cases, which indicates that acknowledging that it is highly probable that neighbor sites have the same most likely tree improves breakpoint detection.

### Ability to detect the breakpoint position

**Figure 4:**
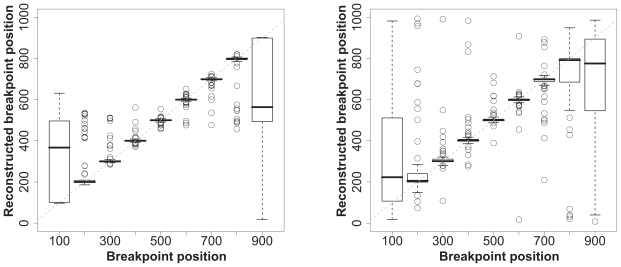
Ability of the Phylo-HMM (left) and Mixture Model (right) to detect the breakpoint position in simulated alignments. The dashed grey line corresponds to values that would be obtained with an ideal method, whose reconstructions are identical to simulations.

Both the MM and the Phylo-HMM most often detect two segments in the alignment. In such cases, Figure 4 shows that the precision with which the breakpoint is predicted displays the same dependency upon the length of the smaller segment as the ability of the models to detect the number of segments. The phylo-HMM seems slightly better than the MM in detecting the precise breakpoint position when the smallest partition is ≥200 bases long. Although the Phylo-HMM seems not as good as the Mixture Model when the smallest partition is 100 bases long, the difference between the two methods is not significant (Student t-test and Wilcoxon test on the absolute differences between expected position and predicted position). This suggests that using more than a single autocorrelation parameter in the HMM method may not be useful, even when segment lengths are very dissimilar.

### Ability to recover the true topologies

**Figure 5:**
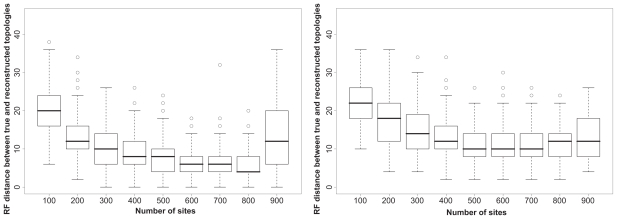
Ability of the Phylo-HMM (left) and Mixture Model (right) to recover topologies from simulated alignments. RF distances were computed between simulated and reconstructed trees for each part of the alignments, and are reported with respect to the number of sites the reconstructed trees are based upon.

On average, the Phylo-HMM is better at recovering the trees used in the simulation than the MM, and both models find it easier to get good trees if the alignment that has been simulated along them is long (Fig. 5). However, the quality of the reconstructed trees finds an optimum for alignments that are 600 to 800 sites, not longer. When one of the two topologies found in the alignment represents only 100 sites, both topologies, the one found in 100 sites and the one found in 900 sites, are less well reconstructed. We note that the topological accuracy of our algorithm is in line with results obtained by PhyML on alignments 500 bases long,^15^ where the RF distance was reported to be approximately 8 when simulations incorporated rate heterogeneity, as in ours.

### Computation times

Computations were run on the IN2P3 computing centre, on single processors ranging from 2.2 to 2.8 GHz. It took on average 9 min 48 s for the Mixture Model implementation to give a result on the simulations, while only 3 min 45 s for the phylo-HMM. The additional optimization of the autocorrelation parameter has not resulted in an increased computational time, but a decrease, perhaps because the HMM ensures that the set of sites pleading for a given topology is more stable throughout the tree space search than when the MM is used. However, both models are very efficient on datasets containing 40 sequences and on single desktop computers.

### Conclusions on the simulations

Overall, the Phylo-HMM is better able to uncover the recombination structure of simulated alignments, since it more often finds the right number of segments, is more accurate at pinpointing the recombination breakpoint, and also recovers trees closer to the true trees. This is probably because the HMM takes into account the dependency of neighboring sites. Although the HMM approach is superior to the MM approach, we recommend using both the MM and the Phylo-HMM to analyse datasets, and use the MM to confirm or question results obtained with the HMM.

### Application to real protein sequences

Several studies have unveiled recombination events in viruses, for instance in HIV viruses. In 1999, Gao et al discovered that a recombination event in a chimpanzee host was at the origin of the YBF30 (group N) HIV-1 virus: the begining of the genome of YBF30 was most closely related to group M whereas the rest of its genome was most closely related to a chimpanzee virus, SIVcpzUS. They based this conclusion on first a sliding window analysis where divergence between pairs of sequences was computed, and second the reconstruction of trees for two portions of the alignment, on each side of a putative recombination breakpoint, which had been identified by eye. Likelihood tests confirmed the recombination event, showing that the first part of the alignment rejected the tree obtained for the second part, and *vice-versa.*

**Figure 6:**
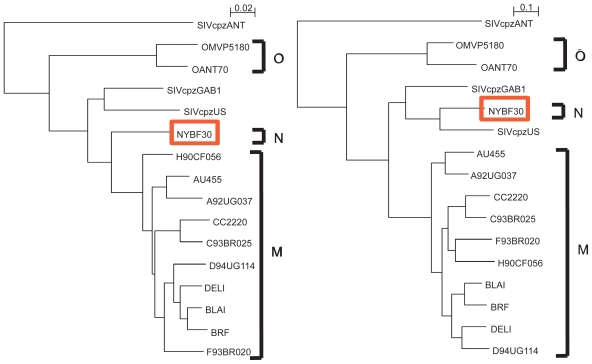
Trees found by the Phylo-HMM on Gao et al data. The trees found by the Mixture model are nearly identical.

This study therefore provides a good test of the ability of the Mixture Model and the Phylo-HMM to detect recombination in natural conditions, on an amino-acid alignment. The two models were run on the alignment from Gao et al, setting the number of trees to two. The Mixture Model predicted two breakpoints, one at position 95, and the other at position 1354. The phylo-HMM predicted only one breakpoint, at position 1353. The two models therefore agree on the presence of a breakpoint around position 1353, which falls very close to the recombination breakpoint determined by eye in the original analysis, at position 1400. The additional breakpoint predicted by the MM is more uncertain as it is not detected by the Phylo-HMM. Interestingly, both the MM and the Phylo-HMM uncover the shifting position of YBF30, which first is close to group M sequences, and then close to SIVcpzUS (see Fig. 6 for trees found with the Phylo-HMM).

This example shows that the Phylo-HMM is also efficient on real sequence datasets. The use of such a program offers an improvement over the sliding-window approach taken by Gao et al; indeed, if one is to look for a recombination event in any sequence, all sequences are to be analysed two by two, which, for the 16 sequences present in the tree amounts to looking at 16 * 15/2 = 240 plots of divergence. With programs such as ours, only two steps are required, as advocated by Chan et al^24^ first a statistical measure to detect the occurence of recombination needs to be applied; if positive, our programs can then be used to precisely pinpoint the recombination breakpoint and reconstruct phylogenetic trees. This way, all the sequences are analysed at once, and the user input is minimal. Eventually, statistical tests such as implemented in Consel^25^ can be applied to confirm the occurence of recombination.

## Improvements

Our approaches are simple and therefore more efficient than sophisticated Bayesian approaches, and can pinpoint recombination breakpoints more precisely than approaches based on sliding windows. Our models however could be improved; importantly, including a model of dependency between topologies before and after a recombination breakpoint may be very useful. Indeed, a recombination event should not entirely change a phylogeny but on the contrary merely change the particular position of a clade. Therefore, on each side of a recombination breakpoint, one could allow only pairs of trees that differ by the position of a single clade. This has been done very recently in the Bayesian framework;^26^ importing this in a maximum likelihood framework while keeping computational efficiency would be an interesting challenge.

## Conclusion

In this article, a Mixture Model and a Phylogenetic Hidden Markov Model to detect recombination were presented. Both methods were tested on synthetic datasets, which showed that the Phylo-HMM was superior to the Mixture Model in most circumstances. Notably, both methods were highly efficient. The analysis of an already published HIV dataset showed that the models could successfully uncover recombination breakpoints and topologies. Future improvements might include searching for the appropriate number of topologies to use, or constraining the topologies on each side of a breakpoint to differ by no more than one rearrangement.
